# Designing and evaluating an interprofessional shared decision-making and goal-setting decision aid for patients with diabetes in clinical care - systematic decision aid development and study protocol

**DOI:** 10.1186/1748-5908-9-16

**Published:** 2014-01-22

**Authors:** Catherine H Yu, Dawn Stacey, Joanna Sale, Susan Hall, David M Kaplan, Noah Ivers, Jeremy Rezmovitz, Fok-Han Leung, Baiju R Shah, Sharon E Straus

**Affiliations:** 1 Keenan Research Centre, Li Ka Shing Knowledge Institute of St. Michael’s Hospital, Toronto, Canada; 2 Department of Medicine, University of Toronto, Toronto, Canada; 3 Dhalla Lana School of Public Health, University of Toronto, Toronto, Canada; 4 School of Nursing, University of Ottawa, Ottawa, Ontario, Canada; 5 Clinical Epidemiology Program, Ottawa Hospital Research Institute, Ottawa, Canada; 6 Mobility Program Clinical Research Unit, Li Ka Shing Knowledge Institute, St. Michael’s Hospital, Toronto, Canada; 7 Institute of Health Policy, Management and Evaluation, University of Toronto, Toronto, Canada; 8 North York Family Health Team, Toronto, Canada; 9 Department of Family and Community Medicine, University of Toronto, Toronto, Canada; 10 Family Practice Health Centre, Women’s College Hospital, Toronto, Canada; 11 Institute for Health System Solutions and Virtual Care, Women’s College Hospital, Toronto, Canada; 12 Institute for Clinical Evaluative Sciences, Toronto, Canada; 13 Sunnybrook Research Institute, Sunnybrook Health Sciences Centre, Toronto, Canada

**Keywords:** Shared decision-making, Priority setting, Patient decision aid, Interprofessional care, Diabetes mellitus, Patient education, Medical informatics, Toolkit development, Study protocol, User-centred design, Qualitative methods

## Abstract

**Background:**

Care of patients with diabetes often occurs in the context of other chronic illness. Competing disease priorities and competing patient-physician priorities present challenges in the provision of care for the complex patient. Guideline implementation interventions to date do not acknowledge these intricacies of clinical practice. As a result, patients and providers are left overwhelmed and paralyzed by the sheer volume of recommendations and tasks. An individualized approach to the patient with diabetes and multiple comorbid conditions using shared decision-making (SDM) and goal setting has been advocated as a patient-centred approach that may facilitate prioritization of treatment options. Furthermore, incorporating interprofessional integration into practice may overcome barriers to implementation. However, these strategies have not been taken up extensively in clinical practice.

**Objectives:**

To systematically develop and test an interprofessional SDM and goal-setting toolkit for patients with diabetes and other chronic diseases, following the Knowledge to Action framework.

**Methods:**

1. Feasibility study: Individual interviews with primary care physicians, nurses, dietitians, pharmacists, and patients with diabetes will be conducted, exploring their experiences with shared decision-making and priority-setting, including facilitators and barriers, the relevance of a decision aid and toolkit for priority-setting, and how best to integrate it into practice.

2. Toolkit development: Based on this data, an evidence-based multi-component SDM toolkit will be developed. The toolkit will be reviewed by content experts (primary care, endocrinology, geriatricians, nurses, dietitians, pharmacists, patients) for accuracy and comprehensiveness.

3. Heuristic evaluation: A human factors engineer will review the toolkit and identify, list and categorize usability issues by severity.

4. Usability testing: This will be done using cognitive task analysis.

5. Iterative refinement: Throughout the development process, the toolkit will be refined through several iterative cycles of feedback and redesign.

**Discussion:**

Interprofessional shared decision-making regarding priority-setting with the use of a decision aid toolkit may help prioritize care of individuals with multiple comorbid conditions. Adhering to principles of user-centered design, we will develop and refine a toolkit to assess the feasibility of this approach.

## Background

Prevention of diabetes complications requires a multitude of self-management tasks which are time-consuming. For example, following the American Diabetes Association recommendations for self-management would take 143 minutes per day [1]. Patients spend a mean of 58 minutes per day on self-care, with the largest barrier to additional care being ‘not enough time’ [1]. To compound this, the majority (56%) of people with diabetes have two or more additional chronic conditions [2]. Patient adherence to clinical practice guideline (CPG) recommendations is impacted by multi-morbidity, as it directly impacts self-management ability (*e.g*., depression) [3] and competes for time and attention [4] (Yu CH: Impact of a web-based self-management intervention for patients with type 2 diabetes on self-efficacy, self-care and diabetes distress, submitted). For example, patients with a greater overall number of comorbidities placed lower priority on diabetes, had worse diabetes self-management ability [5,6] and poor cardiometabolic control [7]. Patients with a greater number of comorbidities also valued longevity and quality of life (*e.g*., reduction in complications) [8,9]. In contrast, primary care physicians prioritized glycemic control, blood pressure control, and lipid lowering over interventions such as retinal and renal screening [10]. In one study where primary care physicians were mailed a clinical vignette of a patient with diabetes whose main issue was worsening chronic low back pain, only 44% of physicians prioritized pain as the primary issue [11]. Thus, competing disease priorities and competing patient-physician priorities present challenges in the provision of care for the complex patient.

To help patients and providers reach goals in managing multiple risk factors, effective shared decision-making (SDM) tools, or decision aids, are needed to facilitate prioritization of treatment options. SDM consists of patients and providers establishing an ongoing partnership in exchanging information; deliberating on options; and deciding upon the priority for taking action and acting on the decision [12]. Though initially developed for use in acute care contexts, it has been adapted for use in chronic care, including diabetes [13]. SDM has the potential to improve patient care. A systematic review of randomized controlled trials of SDM identified 11 studies [14]; interventions included an interactive video program, question/information sheet, or card-ranking discussion. Of the five studies that examined physical and psychological wellbeing, two studies reported positive outcomes; both were characterized by long-term decisions that occurred over more than one session in the setting of chronic disease, thereby indicating a role for SDM in complex diabetes care.

Mediating factors to the successful implementation of SDM have been reported. In a review of 38 studies [15], the most common barriers were time constraints, and lack of applicability due to patient characteristics and his/her clinical situation. Facilitators of SDM included provider motivation, having a positive impact on clinical process and outcome [16], healthcare provider training, and patient-mediated interventions [17]. Specific to diabetes, patients have reported that patient/provider power imbalance, health literacy and denial of the condition were barriers to SDM. Provision of medical knowledge, validation of patient experiences, strong interpersonal skills, and provider availability were facilitators of SDM [18].

These barriers to SDM can be overcome by incorporating IP care providers. Furthermore, diabetes care may occur in the context of interprofessional (IP) care [19], defined as promotion of high-quality care through synergistic efforts of multiple healthcare professionals’ [20-23]. Several systematic reviews in diabetes care have shown that role expansion, active participation by more than one discipline, and the addition of new healthcare team members [24-26] improve clinical outcomes. In addition to improving clinical parameters, incorporating an IP approach may facilitate uptake of SDM [27].

SDM can be facilitated by the use of patient decision aids (PtDAs) [17,28,29], as they help frame the decision to be made. A 2011 Cochrane review of PtDAs [30] identified 86 studies and found that PtDAs improved decision quality and process, and that detailed PtDAs were more effective than simpler PtDAs [31]. Furthermore, when the PtDA was used within the consultation with the healthcare practitioner, patients were more likely to achieve SDM with their practitioner when compared to patients using a PtDA on their own [31,32]. In a search of the published literature, we identified four PtDAs focusing on diabetes, one evaluated with a prospective observational study [33], and three evaluated through randomized controlled trials [34-37]. These PtDAs included a goal-setting intervention [33], ‘Diabetes Medication Choice’ [38], ‘Statin Choice’ [34-36,39], and a ‘Metabolic control’ aid [37]. These were studied in specialty clinics [33-36], primary care [38,39] and general public [40]. While knowledge, risk perception, satisfaction, decision-making participation, trust, decisional conflict, and documented goals improved, there was no impact on diabetes empowerment nor clinical outcomes; effect on adherence was mixed. With the exception of one tool [33], these PtDAs focused on one specific decision (such as the decision to initiate lipid-lowering therapy) and did not specifically address patient-important priorities and decisions. However, potential exists for their use in setting healthcare goals and prioritizing disease management strategies in patients with diabetes and other comorbidities [33]. Corser *et al*. (2007) has set the precedent with a brief SDM goal-setting intervention in an American primary care clinic [33]. This intervention consisted of a 28-page patient workbook, a 2-hour patient education session and two 2-hour medical resident seminars. Post-intervention knowledge increased significantly (P = 0.001), as did a number of documented diabetes goals (pre: 0.67 goals; post: 1.09 goals; P <0.001). Glycemic control, weight, and diabetes empowerment scores showed a trend toward improvement. While this study was limited by its observational nature, it suggests a role for SDM in goal-setting in patients with diabetes. We anticipate that effectiveness can be further optimized with the additional provision of a point-of-care tool for use at the time of consultation and a provider-specific tool, to be used longitudinally, and with more explicit integration of the interprofessional team.

We hypothesize that a multi-component PtDA toolkit (patient-directed, provider-directed and point-of-care tools) that individualizes care priorities and incorporates an IP approach to SDM may help to prioritize complex guideline recommendations for patients with type 1 or type 2 diabetes and other comorbidities. Therefore, such a tool may improve the relevant uptake of the Canadian Diabetes Association (CDA) 2013 CPG [41] into practice.

## Methods

This study will be guided by the Knowledge to Action (KTA) framework [42] to develop, test and refine a multi-component IP-SDM toolkit designed to help prioritize guideline-based disease management in patients with multimorbidity, defined as those with type 1 or type 2 diabetes and additional chronic conditions. In the KTA framework, knowledge is created, refined, then applied through seven iterative steps. This model is appropriate for the systematic development and evaluation of our intervention as it allows for close integration of researchers and knowledge-users, systematic and iterative intervention development, rigorous evaluation and sustainability. In this protocol, we outline the first four steps (Table 1). We also integrated the Medical Research Council framework for the development and evaluation of complex interventions [42], as it provides additional rigour regarding complex interventions, defined as those that entail numerous interacting components, numerous behaviours required, numerous groups targeted, or numerous outcomes [42]. This combined model is appropriate for the systematic development and evaluation of this intervention [43].

**Table 1:** Knowledge to Action framework

**Steps**	**KTA framework**	**Relevance to study proposal**
1	Identify review and select knowledge	Knowledge has been refined into a high quality CPG [41], and there is an evidence to practice gap (Background). We have identified the existing evidence regarding individualization of care and SDM in an IP context, and the IP-SDM framework on which to build our intervention (Background).
	Identify problem	
2 & 3	Adapt knowledge to local context	The MOHLTC’s Ontario Diabetes Strategy aimed to improve the care of Ontarians with diabetes through access to technology, specialized resources and health professionals, to enable patients and providers to actively co-manage, monitor and improve care. New education teams (nurse educators and dietitians) were created to help support people with diabetes in primary care; thus our use of the IP-SDM framework complements this context. Having identified barriers to CPG and SDM uptake (Background) in the literature, we will identify barriers in our *targeted* population by conducting **(Phase 1)**, the feasibility substudy (Methods).
	Assess barriers to knowledge use	
4	Select, develop and tailor the intervention	Based on potential facilitators of SDM adoption identified in the literature [17] (Background), a previously reported goal-setting intervention [33] (Discussion) and the findings of Substudy 1, our IP-SDM intervention will be developed **(Phase 2)**. Subsequently, we will then conduct heuristic evaluation **(Phase 3)** and usability testing **(Phase 4)**.

### Study overview

The study detailed in this protocol paper consists of four phases: feasibility testing (Phase 1); toolkit development (Phase 2); heuristic evaluation (Phase 3); and usability testing (Phase 4). Throughout the development process, the toolkit will be refined iteratively based on findings of each phase.

This program of research will continue with a rigorous evaluation of the effectiveness of the PtDA toolkit. Our intent is to conduct a two-step clustered RCT (Phase 5).

### Phase 1. Feasibility testing

This phase will consist of assessing the feasibility of IP-SDM. Individual semi-structured interviews with healthcare providers and patients (60 to 75 minutes long) will be used to explore their experiences with shared decision-making and priority setting, including facilitators and barriers, the relevance of a decision aid and toolkit for priority-setting, how best it would be integrated into practice and potential content of the PtDA [44]. After exploring these concepts, participants will work through a prototypic PtDA (Additional file 1), setting priorities for diabetes management with the interviewer providing scripted responses as the ‘patient’ or ‘provider.’

### Participants

A purposive sampling strategy will be adopted to ensure sample heterogeneity (in order to capture the perspectives of healthcare providers and patients with varied clinical and life experiences) [45].

A total of 20 family physicians with varied socio-demographic profiles (age, gender, remuneration plan, academic vs. community) and 13 to 15 nurses, dietitians and/or pharmacists (either certified diabetes educators or not) with varied profiles (age, gender, academic vs. community) will be recruited through family health teams in the academic and community settings in the Greater Toronto Area.

A total of 13 to 15 patients with type 1 or type 2 diabetes and two other comorbidities (heart disease [including ischemic, valvular, congestive, arrhythmic, congenital disease]; stroke; hypertension; cancer [excluding non-melanoma skin cancer]; chronic lung disease; arthritis, inflammatory bowel disorders; urinary incontinence) with varied socio-demographic profiles (gender, educational attainment) in these Family Health Teams will be identified through their healthcare providers or chart review. We selected these conditions because of their chronic nature, high prevalence [2] and impact on self-management. Patients who do not speak English or who are unable to provide consent will be excluded. Women who are pregnant or considering conception will be excluded.

A sample size of 13 participants per group (10 initial interviews with 3 subsequent interviews to ensure no new themes emerging) will likely be necessary to achieve saturation and to generate sufficient feedback to refine the toolkit [46]. We are proposing to recruit a greater number of physicians to account for additional demographic strata as mentioned above.

### Data collection

One-on-one interviews with patients and providers will be based on interview guides developed by team members with expertise in qualitative research and SDM. We will focus on mediators to PtDA adoption and appropriateness of content and formats used (Additional file 2: Phase 1 Interview Guide). Healthcare provider interviews will be conducted at their practice site or by telephone; patient interviews will be conducted at our research site or by telephone. We will use open-ended questions based on pre-existing literature regarding barriers and facilitators to SDM and goal-setting [17,18] and the Theoretical Domains framework [40]. This framework includes constructs from 33 behavior change theories and was developed to make theories more accessible to implementation researchers. We selected this approach because it has good construct validity and has been widely applied in healthcare implementation research [47]. The participant will also use a decision-aid prototype, created by the principal investigator with content from evidence-based guidelines [41] and an International Patient Decision Aid Standards checklist [48] with the interviewer substituting the role of the healthcare provider for patient interviews or patient for healthcare provider interviews with scripted responses. Face validity of this guide will be assessed with other team members and refined as needed; we will test the instrument on team members, including knowledge user team members (such as primary care physicians, nurse, and person with diabetes) before conducting interviews. A trained interviewer will conduct each interview session. All individual interviews will be audio taped and field notes will be kept of all interviews.

### Analysis

Audiotapes will be transcribed to create a verbatim transcript. In keeping with qualitative methodology, data analysis will occur in conjunction with data collection. Ongoing analysis will inform the development of progressive iterations of the interview guides. Inductive thematic analytic techniques will be employed [49-51]. Transcripts will be coded for emergent categories and themes within and across interviews [44]. All transcripts will be coded and reviewed independently by two team members with expertise in qualitative research methods. Consensus on coding will be reached through comparison and discussion among these reviews. Memos kept by the qualitative team members will help monitor the analysis, and regular meetings will enable continued dialogue and discussion within the project. NVivo software (v.10) will be used for analysis.

### Phase 2. Toolkit development

Based on potential facilitators of SDM adoption identified in the literature [17], a previously reported goal-setting intervention [33] and the findings of Phase 1, the IP-SDM toolkit will be developed.

Development of the toolkit will rely on the IP-SDM framework [52] and follow the International Patient Decision Aids Standards criteria [48]. The International Patient Decision Aids Standards provide explicit guidance on content, development process, and effectiveness. The IP-SDM framework consists of seven steps in SDM and considers the role of the patient, the family and the healthcare team [53]. Content of our toolkit will include: eliciting the patient’s general priorities of care; eliciting his/her diabetes-specific goals and outcomes; outlining diabetes-specific therapies and detailing population-specific benefits and risks. We will select the content based on the evidence underlying the CPG recommendation as well as patient values elucidated in Phase 1. Use of the IP-SDM toolkit within the team will be fluid and dependent on the usual roles, responsibilities and processes of care and the needs of the patient. The initial IP-SDM toolkit will be in the English language; the patient-directed components will target a Grade 8 literacy level (readability score) [48].

Following development, the PtDA will be reviewed by expert healthcare providers and patients not involved in the development process for content accuracy. The research team will identify two each of family physicians, endocrinologists, geriatricians, nurses, dietitians, pharmacists and patients. Reviewers will complete a report to determine the PtDA’s accuracy, comprehensiveness, balance of perspective, and ease of understanding (Additional file 3). Data from the report form will be collated and analyzed descriptively; any discordant responses between reviewers will be discussed and resolved by the research team. Revisions to the toolkit content will be made based on this feedback.

### Phase 3. Heuristic evaluation

This phase will consist of heuristic evaluation, in which a human factors engineer, following the methodology set out by Nielsen [37,51,53,54], will review the PtDA. Usability issues will be identified, listed, then categorized by severity into minor, moderate, major or catastrophic; severity estimates will be based on frequency, impact and persistence of errors.

Based on these findings, the IP-SDM toolkit will be refined in an iterative fashion by study team members, including a graphics designer and computer programmer, as necessary.

### Phase 4. Usability testing

Usability testing using cognitive task analysis [55], whereby users are individually asked to ‘think aloud’ as they perform specific tasks to cover the major functionalities of the IP-SDM toolkit (provider enabler, PtDA and patient workbook) will be conducted in a 60- to 75-minute session.

### Participants

A consecutive sample of 16 patient-provider dyads will be invited to participate. We will first recruit health care providers (eight primary care physicians, and eight nurses, dietitians and/or pharmacists). We will use a convenience sample of healthcare providers of varied socio-demographic profiles (described in Phase 1) recruited from family practice units at academic health science and community centers in the Greater Toronto Area. We will ask each provider participant to identify a patient with diabetes who will be invited by our research coordinator. We will try to ensure a sampling of patients with type 1 or type 2 diabetes and two other comorbidities with varied socio-demographic profiles (described in Phase 1). Research has shown that up to 80% of usability issues can be identified through five to eight participants [55]. We anticipate conducting up to three usability cycles of four to five participant dyads in a process of iterative redesign.

### Data collection

A consultant with expertise in health informatics and human factors engineering will conduct each session in the primary care or research setting. After a brief overview of the process, the consultant will prompt users with a structured interview guide with regards to usability. Paths users take to accomplish tasks, errors made, when and where they encountered confusion or frustration, and time spent on the PtDA toolkit or individual tasks within the PtDA toolkit will be documented. Following completion of the task, participants will be interviewed regarding degree of satisfaction, strengths and weaknesses of the toolkit, and the quality of decision support given by the provider to the patient. All interviews will be audio-taped. Field notes will be kept of all sessions as a further source of data for analysis.

### Analysis

Data analysis will be conducted as described in Phase 1. Audiotapes will be analyzed independently by two members of the research team to assess knowledge use (*i.e.*, did SDM occur for each patient-provider dyad) using a validated scale, the Decision Support Analysis Tool (DSAT-10) [56]. DSAT-10 evaluates practitioners’ use of decision support during a clinical encounter. It consists of six categories of decision support skills and four categories of communication skills. The overall inter-rater agreement and kappa coefficients were, respectively 75% and 0.59 for the decision support skills, and 76% and 0.68 for the communication skills. Disagreement will be resolved by discussion. A descriptive analysis of the numerical data will be performed.

Based on these findings, the IP-SDM toolkit will be refined in an iterative fashion, after four to five interviews.

### Research ethics

The study was approved by the Research Ethics Boards of St. Michael’s Hospital (reference number 13-014C), Sunnybrook Health Sciences Health Centre (reference number 345–2013) and Women’s College Hospital (reference number 2013–0058).

### Study status

We are commencing recruitment at study sites.

## Discussion

Diabetes is a complex disease, often associated with multiple other conditions. Competing disease priorities and competing patient-physician priorities present challenges in the provision of care for the complex patient, thereby reducing guideline adherence. Adopting a patient-centered SDM model of care, wherein the patient sets goals and management priorities with the support of healthcare providers, has the potential to improve uptake of patient-relevant guideline recommendations, improve the process of care and patient-important outcomes such as quality of life. Our objective is to systematically develop, test and refine a diabetes-focused SDM and priority-setting PtDA following the KTA framework [32].

While SDM and the use of PtDAs hold promise, successful implementation would require good quality PtDAs targeting the users’ needs with careful attention to development [48], effective mechanisms and formats to deliver it in clinical care [30], and models to support their implementation in clinical practice [31]. We will address these factors in our project.

Study strengths include our systematic evidence-based and user-centered development and implementation, enabled by team expertise and knowledge-user engagement. The team includes expertise in shared decision-making, knowledge translation, information technology, primary care diabetes, and qualitative and quantitative research methods, as well as key knowledge users – primary care providers and people with diabetes.

Study limitations include potential volunteer bias and recruitment. To address volunteer bias, we will compare socio-demographic characteristics of participants and non-participants. Successful recruitment and implementation will depend on strong knowledge user engagement, which we have developed by creating a combined knowledge user-researcher team.

Competing health concerns present real obstacles to people living with diabetes and other chronic disease and their primary care providers. Guideline implementation interventions to date do not acknowledge these impediments, and as a result, patients and providers are left overwhelmed by the sheer volume of recommendations. Shared decision-making regarding priority-setting with the use of PtDAs may help focus care of these individuals. An interprofessional approach to SDM may also overcome the barriers to implementation of the PtDA such as time, availability and perceived power imbalance. Adhering to principles of user-centered design, this toolkit will be developed and refined to assess the feasibility of this approach. Given the growing prevalence of diabetes and multimorbidity, effectively bridging the knowledge to practice gap in this area has the potential to significantly improve patient-important outcomes, healthcare delivery and system sustainability.

## Abbreviations

CPG: Clinical practice guidelines; SDM: Shared decision making; PtDAs: Patient decision aids; IP: Interprofessional; CDA: Canadian Diabetes Association; KTA: Knowledge to action.

## Competing interests

The authors declare that they have no competing interests.

## Authors' contributions

CY conceived of the study and drafted the manuscript. All authors participated in the design of the study, revised the manuscript critically for intellectual content, and have read and approved the final manuscript.

## Supplementary Material

Additional file 1 **Data: Prototypic decision aid.** Point of care worksheet, with sample information booklet pages and interactive wheel chart. Click here for file

Additional file 2 **Data: Phase 1 Interview Guide.** Script of semi-structured interviews. Click here for file

Additional file 3 **Data: Peer review report form.** Report to be completed by expert reviewers. Click here for file
