# Supplementary material for: Designing and evaluating an interprofessional shared decision-making and goal-setting decision aid for patients with diabetes in clinical care - systematic decision aid development and study protocol
Source: Implement Sci. 2014 Jan 22;9:16. doi: 10.1186/1748-5908-9-16 (PMC3937124; doi:10.1186/1748-5908-9-16)

Appendix 1: Prototypic decision aid (Point of care worksheet, with sample information booklet pages and interactive wheel chart) (page 1/2)

A worksheet to help you pick diabetes goals that are important to you

- Diabetes can be a complicated disease that can keep you and your loved ones busy!
- This worksheet, and the information booklet that comes with it, can help you and your health care team pick goals that are important to you and strategies to help you get there.
- With time, your priorities can change, so you can come back to this worksheet again and again!

First, a little bit about you…

- How beneficial treatments are to you depends on your health. Please fill in the next 3 sentences about yourself:

1. I am _____ years old.
2. I have had diabetes for _____years
3. I have / have not (circle one) had a heart attack, stroke or blood vessel narrowing.

What goals are important to you?

- Which **general** health goals are important to you? (check all that apply)

❑Live a long life
❑Maintain good function
❑Prevent or decrease symptoms

- If you have checked more than 1, try to think of which is the **most** important to you and **circle** this one)

What goals are important to you?

- What **diabetes** health goals are important to you? (check all that apply)

❑Prevent heart disease
❑Prevent stroke
❑Prevent foot ulcers & amputation
❑Prevent blindness
❑Prevent kidney disease
❑Prevent nerve damage

- If you have checked more than 1, try to think of which is the **most** important to you and **circle** this one

There are many things that you can do to reach these goals:

❑Connect with your health care team
❑Get blood and urine tests
❑Stop smoking
❑Control your blood sugars
❑Lower your blood pressure
❑Lower your cholesterol
❑Take ASA
❑Check your feet

- The benefits & downsides of each of these are described in the information booklet that comes with this worksheet; the tabs are coded with the symbol of the goal you are interested in.

The strategy that you pick can achieve more than one goal

- For example, choosing to lower your blood pressure may help you
  - Live a long life
  - Maintain your activities
  - Prevent heart disease
  - Prevent stroke
  - Prevent blindness
  - Prevent kidney failure

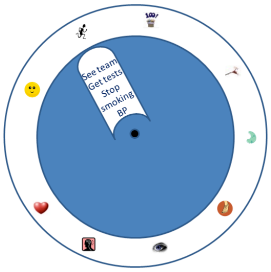

	General Goals			Diabetes-Specific Goals					
Diabetes-specific management strategies (sub-heading for each = recs)	Live a long life	Maintain function	Prevent or reduce symptoms	Prevent heart disease	Prevent stroke	Prevent foot ulcers & amputation	Prevent blindness	Prevent kidney failure	Prevent nerve damage
See my health care team		✓	✓			✓	✓		
Get blood and urine tests	✓	✓	✓					✓	
Check my feet						✓			
Stop smoking	✓	✓	✓	✓	✓	✓			
Lower my blood sugars - Diet/ex/weight/check/med	✓		✓	✓			✓	✓	✓
Lower my blood pressure - Diet/ex/weight/med/check/ ACEiARB	✓	✓	✓	✓	✓		✓	✓	
Lower my cholesterol - Diet/ex/weight/med/Statin	✓	✓	✓	✓	✓		✓		
Take aspirin									

And here's how diet, exercise, weight loss and taking medications can help with your goals:

	Lower blood pressure	Lower cholesterol	Lower blood glucose	Lose weight
**Diet**	✓	✓	✓	✓
**Exercise**	✓	✓	✓	✓
**Lose weight**	✓	✓	✓	
**Take medications**	✓	✓	✓	

Which of the following goals are most important to you today?

- (check all that apply)

❑Live a long life
❑Maintain good function
❑Prevent or decrease symptoms
❑Prevent heart disease
❑Prevent stroke
❑Prevent foot ulcers & amputation
❑Prevent blindness
❑Prevent kidney disease
❑Prevent nerve damage
❑None

What strategies do you want to do now?

Based on your goals, and balancing the benefits and downsides described in the booklet, what strategy do you want to do now?

❑Connect with your health care team
❑Get blood and urine tests
❑Stop smoking
❑Control your blood sugars
❑Lower your blood pressure
❑Lower your cholesterol
❑Take ASA
❑Check your feet

Now you're ready to set your goals

My goal	Where I am now	Where I want to be
Prevent a stroke	Had a mini-stroke 2 months ago	Prevent another stroke
Lower my blood pressure	145/92	Lower my blood pressure to < 130/80

What strategies are you going to use?

In __3__ month(s): (pick a time frame)

How I'm going to reach my goal	What I'm doing now	What I will do
Hint: Look at the summary table, or refresh your memory by reviewing your information booklet.		
Take ASA	Not yet taking ASA	Take 81 mg of ASA every day
Lower my blood pressure to < 130/80	Not adding salt<br>Already taking ramipril 5 mg	Cut out ham<br>Increase ramipril to 10 mg every day

Appendix 1: Prototypic decision aid (Point of care worksheet, with sample information booklet pages and interactive wheel chart) (page 2/2)

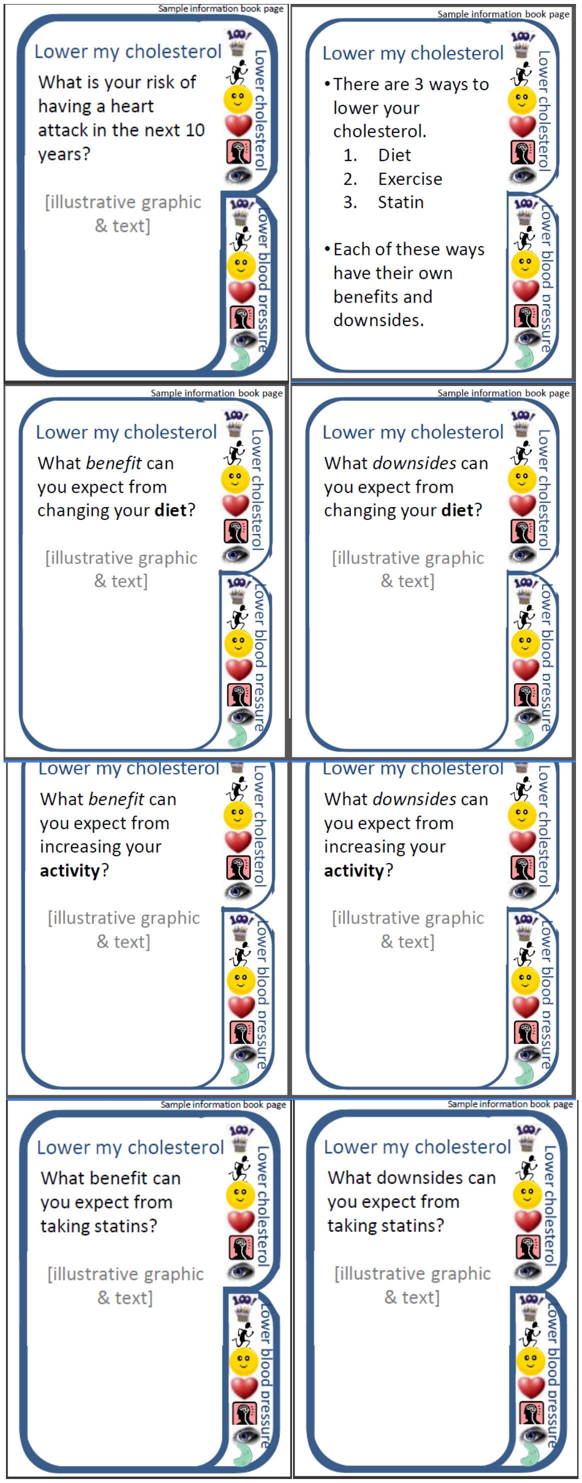

Supplement: Additional file 1 — Data: Prototypic decision aid. Point of care worksheet, with sample information booklet pages and interactive wheel chart. [file 1748-5908-9-16-S1.pdf]
