# Supplementary material for: Designing and evaluating an interprofessional shared decision-making and goal-setting decision aid for patients with diabetes in clinical care - systematic decision aid development and study protocol
Source: Implement Sci. 2014 Jan 22;9:16. doi: 10.1186/1748-5908-9-16 (PMC3937124; doi:10.1186/1748-5908-9-16)
Supplement: Additional file 2 — Data: Phase 1 Interview Guide. Script of semi-structured interviews. [file 1748-5908-9-16-S2.pdf]

Appendix 2: Phase 1 Interview Guide

Thank you for taking the time to meet with me today. My name is_____________ and I work with_____________ from _____________. We are conducting interviews with health care professionals and patients to help us understand how you prioritize your patients' health care needs for managing diabetes and other chronic diseases.

Specifically we will discuss your experiences with patients and explore how you optimize their diabetes management. Findings from these interviews will help us design an aid to help patients make decisions about their diabetes.

The interview should take about 45 to 60 minutes. I will be taping the interview so that I don't miss any of your comments. All of your comments will remain confidential. You don't have to respond to any question that makes you uncomfortable.

Have you had a chance to read the consent form? Do you have any questions about the consent or about what I have just explained to you? Are you willing to participate?

Great, let's start.

1. What is your usual approach to making decisions such as treatment and diagnosis decisions around diabetes? (e.g. make the decision for the patient, share the decision with the patient, provide support or advice for patients to make the decision on their own)
   PROBES
   - [If answers "depends"]: In which situations would you:
     - Make the decision for the patient?
     - Share the decision with the patient?
     - Provide support or advice for patients to make the decision on their own?
   - [Related]: For which health care decisions should a patient be actively involved? (examples: getting stress test, starting a statin)
   - How do you think your patients usually go about making decisions about their health care?
   - Think back to over the last 2 weeks. Can you describe an ideal situation where you made a decision with a patient in your practice? What decision were you and your patient trying reach?
   - IF SDM HAS NEVER BEEN USED – WHAT DO YOU KNOW ABOUT THE BENEFITS OF USING SDM IN PRACTICE?

2. How do you set priorities about a patient's care when treating a patient with diabetes who has multiple competing health issues (such as high blood sugars, burning feet, high blood pressure?
   PROBES
   - What are the steps that you go through as you set priorities for care?

- How do you currently accommodate your patients' goals for their health care in your practice?
- How do a patient's goals, either for their health care or their life goals, impact how you set priorities for a patient's care?
- Do you have any tools or strategies you use when setting priorities with patients? (may draw on a paper the different issues to prioritize)
- How could you improve how priorities are set for your patient's health

3. Tell me about something that helped you and your patient set priorities for the patient's health care goals.
   PROBES
   - What factors make it easier for you to support your patients' decision-making?
   -
4. Tell me about the challenges you face in your practice when you are attempting to set priorities for your patient's health care goals.
   PROBES
   - What factors make it difficult for you to support your patients' decision-making?

5. Let's move on to another topic. What type of tool or tools have you used that would help you with setting priorities with patients for managing diabetes?
   PROMPTS
   - A decision aid is a tool, like a workbook or a worksheet, that outlines treatment options and their risks and benefits. Are you aware of patient decision aids?
   - Have you used one? Explain.
   - What is your comfort level with using one?
   - How would the use of a decision-making aid help you in your practice? How would the use of a decision-making aid hinder you in your practice?
   - A goal-setting decision aid that would help patients reflect on and evaluate their health care priorities and goals, and identify methods to reach those goals. How would such a decision aid affect your relationship with your patients? (can refer to tool)
   - [IF ANSWERS "HAS NOT USED TOOLS"]: Why? (e.g. not aware that they are available, aware but didn't think they were relevant nor useful, aware but thought they were cumbersome, no time; can further explore their response)

6. We have a prototype of a patient decision aid that we would like you to take a few minutes to review. It is electronic, not paper based. What do you think about this aid?
   PROMPTS
   - Language?
   - Things missing/things that should be removed?

7. How might you use a patient decision aid like this one in your practice?
   PROMPTS
   - How do you see yourself using such an aid or not

- Who do you think should review this tool with a patient? i.e. Physician, dietician, RN, NP, etc?
- What do you feel about a patient reviewing and completing the aid at home?
- How do you feel about the patient then reviewing the aid: 1) in the clinic with a physician, 2) dietician, 3) RN or 4) NP?
