# Supplementary material for: Designing and evaluating an interprofessional shared decision-making and goal-setting decision aid for patients with diabetes in clinical care - systematic decision aid development and study protocol
Source: Implement Sci. 2014 Jan 22;9:16. doi: 10.1186/1748-5908-9-16 (PMC3937124; doi:10.1186/1748-5908-9-16)
Supplement: Additional file 3 — Data: Peer review report form. Report to be completed by expert reviewers. [file 1748-5908-9-16-S3.pdf]

Appendix 3: Peer review report form

Please review the decision aid and consider the following questions. Upon completion of the review, please complete the report form.

	Strongly Agree		Neutral		Strongly Disagree
As a whole, does the decision aid accurately represent the evidence to date?	1	2	3	4	5

Please list and elaborate on components of the decision aid that do not accurately represent the evidence to date:

____

____

	Strongly Agree		Neutral		Strongly Disagree
As a whole, does the decision aid comprehensively cover all relevant aspects of decision-making and goal-setting in diabetes care?	1	2	3	4	5

Please list and elaborate on components of the decision aid that do not accurately represent the evidence to date:

____

____

	Strongly Agree		Neutral		Strongly Disagree
As a whole, does the decision aid present a balanced perspective of decision-making and goal-setting in diabetes care?	1	2	3	4	5

Please list and elaborate on components of the decision aid that do not present a balanced perspective:

____

____

	Strongly Agree		Neutral		Strongly Disagree
As a whole, is the decision aid easy to understand?	1	2	3	4	5

Please list and elaborate on components of the decision aid that are not easy to understand:

____

____
